# Perspectives on the Development of Filter Media for Point of Use Water Filters: Case Study of Arsenate Removal

**DOI:** 10.3389/fchem.2022.826440

**Published:** 2022-04-01

**Authors:** Samuel Chigome, Dickson Andala, Moses Kabomo, Erick Mobegi

**Affiliations:** ^1^ Nanomaterials Division, Botswana Institute for Technology Research and Innovation, Gaborone, Botswana; ^2^ Department of Chemistry, Multimedia University of Kenya, Nairobi, Kenya; ^3^ Department of Chemistry, University of Botswana, Gaborone, Botswana; ^4^ Department of Chemistry, Kisii University, Kisii, Kenya

**Keywords:** nanocomposite, water filter, point of use, local materials, nanotechnology

## Abstract

The demand for access to clean water will continue to increase as the world population increases. For sustainable development and embracement of technological advancement, it is plausible to consider a filter material development approach that uses locally abundant natural resources as the raw material and nanotechnology techniques for material fabrication. The review and research paper will present a perspective of the authors on how to embrace nanotechnology for filter media development with key focus on the remediation of arsenate. Drinking water contaminated with arsenic is an emerging global challenge. Continuous exposure to drinking water with high levels arsenic could result in several types of cancer. With this in mind, the US EPA in 2001 set 10 ppb as the maximum contaminant level of arsenic from the initial 50 ppb. Therefore, arsenic remediation is key in mitigating these health risks in people residing near water bodies with elevated arsenic levels. Adsorption is considered to be the cheapest. However, from literature, majority of the adsorbents cannot be used in field applications due to challenges associated with low adsorption capacity and a high level of particle leaching into purified water thus posing health dangers. Therefore, it means that many of these adsorbents are economically non-viable. A new chitosan, aluminium, titanium, iron and zirconium (CTS-Al-Ti-Fe-Zr) hybrid was fabricated through the sol-gel process. The material was characterized by scanning electron microscopy, Brunauer–Emmett–Teller and Fourier Transform Infrared spectroscopy before and after adsorption. Batch adsorption properties towards As(V) were separately studied as a function of the effect of adsorbent dose, pH, initial concentration, contact time and competing ions. Characterization results show that the material is a polycrystalline with a specific surface area of 56.4 m^2^g^−1^. Further, FTIR and SEM-EDAX showed adsorption of arsenate on the surface of the nanocomposite. Research findings suggest that with only 100 mg of the adsorbent arsenate can be reduced to less than 10 ppb from an initial concentration of 300 ppb respectively. The maximum adsorption capacity for arsenate removal was recorded as 123 mg/g. The presence of SiO_3_^2-^, CO_3_^2-^, and HCO_3_^−^ ions resulted in a slight decline in the adsorption efficiency of arsenate. The equilibrium data fitted well with the Langmuir isotherm 0.99518. Data from the fabricated prototype Point-of-use filter showed that with 60.0 g of the nanocomposite, it is possible to reduce 650 L of drinking water with an arsenate initial concentration of 300 ppb to less 10 ppb. In conclusion, the research findings suggest that the nanocomposite material is capable of removal of arsenate from contaminated drinking water to WHO acceptable levels with a potential to be up scaled for commercial applications.

## 1 Introduction

It should be noted that about 50% of the global population relies on groundwater as their source of drinking water. However, surface water is continually getting exposed to pollution leaving groundwater as the only viable source However, at present it is not considered as the most secure source since it is increasingly being polluted either by anthropogenic activities or dissolution from natural mineral in the earth’s crust (Bootharaju and Pradeep 2010). Studies have established that underground water sources contain pollutants such as heavy metals like arsenic (Shaji et al., 2021) and anions like fluoride (Adak et al., 2016) among other water contaminants.

Drinking water contaminated with arsenic is an emerging global challenge. It is estimated that about 200 million people across the globe are exposed to drinking water with levels of arsenic exceeding 10 ppb (George et al., 2014). Cases of elevated arsenic levels have been identified in several parts of the world such as Bangladesh, India, Vietnam, Mexico and Argentina. Exposure to arsenic affects renal, heart, dermatologic and nervous system (Tchounwou et al., 2012). A number of studies have reported high mortality rates from various cancers (liver, skin, kidney and bladder) in areas characterized by arsenic pollution (Naujokas et al., 2013).

Typical Arsenic levels in groundwater are estimated to range between 0.5–10 ppb. However, these concentrations rise due to oxidation of arsenic pyrite mineral that consequently leaches arsenic into the water. In natural water the predominant oxidation states are trivalent Arsenite (As (III) and pentavalent arsenate (As (V) (Alispahić et al., 2021).

Arsenic does not exist in Free State in the environment. It is found in combination with Iron and Oxygen. Arsenic has four major oxidation states, +3, +5, −0 and −3 and two different forms: inorganic and organic (Tuutijärvi et al., 2013). Organic forms are rarely present in ground water. However, in natural water the predominant oxidation states are trivalent Arsenite (As (III) and pentavalent arsenate (As (V)) (Alispahić et al., 2021). Usually As (III) is mostly found in anaerobic ground water, whereas As (V), is found in aerobic surface water. In terms of toxicity, As (III) is considered to be about 60 times toxic than As (V) (Herath et al., 2016). WHO has set a maximum of 10 ppb as the threshold of Arsenic in drinking water (Giles et al., 2011).

Sources of Arsenic in groundwater can be categorized as either natural or anthropogenic. Natural source is mainly due to the weathering reactions of arsenic bearing minerals, volcanic activity and biological activity. Anthropogenic sources include smelting of metal ores, percolation of water during mining and use of pesticides and herbicides containing arsenic compounds. Anthropogenic sources contribute to higher levels of Arsenic in water compared to natural sources (Jiang et al., 2013). Typical Arsenic levels in groundwater are estimated to range between 0.5–10 ppb. However, these concentrations rise due to oxidation of arsenic pyrite mineral that consequently leaches arsenic into the water (Shaji et al., 2021).

A number of research activities, particularly in the discipline of nanotechnology, have pointed to the fact that application of nano engineered materials can help in resolving water quality issues by using nanoparticles (Ma et al., 2011). These classes of materials have been found to exhibit improved performance vis-à-vis existing compositions, thereby enhancing their effectiveness in point-of-use water purifiers (Savage and Diallo 2005).

Nanoscale engineered adsorbents have gained great attention in the application in water purification compared to existing bulk adsorbents. However, this class of nanomaterials cannot be used in field applications due to challenges in low adsorption capacity, poor particle separation hence a potential danger in view of their leaching into the purified water. Additionally, most of these nanoadsorbents do not have satisfactory wet strength to stay intact as a granular composition. This characterizes them with poor hydraulic conductivity which leads to excessive pressure drop in the water purification cartridge making their application in filtration devices impossible. Lastly majority of adsorbents used in water purification are in powder form which renders filtration difficult (Kumar et al., 2017). Combining all these limitations, it therefore means that many of these adsorbents are economically non-viable making them ineffective in commercial applications.

Adsorption technique is considered to be the cheapest in terms of cost, maintenance and ease of operation with nanoadsorbents gaining attention in the water purification compared to existing bulk adsorbents. However, some adsorbents cannot be used in field applications due to challenges in low adsorption capacity and high level of particle leaching into purified water thus posing health dangers. Further, this characterizes them with poor hydraulic conductivity which leads to excessive pressure drop in the water purification cartridge making their application in filtration devices impossible. (Kumar et al., 2017). Combining all these limitations, it therefore means that many of these adsorbents are economically non-viable making them ineffective in commercial applications. Therefore, there is need to synthesize nanoadsorbents that can overcome the above limitation and that can be easily up-scaled from the laboratory to commercial scale.

The novelty of this work is underpinned on the overcoming the aforementioned limitations of other adsorbents that have been reported in literature. Whereas other studies have reported higher adsorption capacities many go beyond batch adsorption studies. For instance, the powdered adsorbents cannot be packed into water purification cartridge because filtration will be hampered. Based on this observation chitosan, aluminium, titanium, iron, and zirconium (CTS-Al-Ti-Fe-Zr) is characterized by improved mechanical strength since it exists as a granular nanocomposite which makes filtration easier and that the material is strong mechanically both in dry and wet conditions. An added advantage of this material is its synthesis protocol that is considered cheap and environmental friendly. The only disadvantage of this nanocomposite is that its quaternary hence slightly expensive in terms of the oxides needed for its synthesis.

In the simplest of terms, a point of use water filter is a filter that is employed in the purification of water at the point where it is being used. POU water filters are different from centralized water treatment systems in that they are used at small scale level and purify less amounts of water. POU water filters can be vital in water purification practices, particularly in Africa, where the need for decentralized water purification systems are solutions to key water challenges.

Materials can be classified into five categories according to their chemical composition: metals, semiconductors, polymers, ceramics and composites (Smith 2003). The ability to fabricate large surface area materials with controlled functionality has opened up new possibilities in the development of filter media, particularly enhanced adsorption capacity and miniaturization of filtration devices (Reisner and Pradeep 2015). Therefore, the use of nanotechnology-based techniques to fabricate water filter media can be considered as holding the future for POU water filters to address the current and emerging challenges. A better understanding of techniques that have the ability to fabricate either powdery nanoparticles or nanofibers from the five categories of materials is necessary to have control of advanced water filter media development. It should also be noted that such techniques allow the conversion of conventional bulk filtration materials into superior performing filter media at the nanoscale without changing the chemical composition. This presents a significant benefit to Africa that has got a significant number of natural resources that can possibly be manipulated into nanoscale-based water filtration media (Mulwa and Mariara 2016; Roksana 2016). Although our experimental work on electrospun fibers is not presented in the current contribution, it is plausible to highlight the most practically useful technique for fabricating nanofibrous water filter media which is referred to as electrospinning. This will help the readers to have a broader view of the avenues to explore in their pursuits to develop point of use water filters that rely on locally abundant materials in Africa.

The use of electrospinning, a technique that relies on repulsive electrostatic forces to draw a polymer solution into nanofibers, as a membrane fabrication technique allows the incorporation of filter media either as composites or as multiple layers in a variety of POU water filter formats. In principle; 1) electrospinning allows the fabrication of filter media from all classes of materials into nanofibrous form as long as there is a way of getting the precursor polymer or composite into a solution or a melt, 2) it carries the main benefit of nanoparticles being surface area, it addresses the challenge of powdered filter media as the fibrous network allows ease of tortuous flow of water which reduces the back pressure and making the POU gravity driven, 3) there is also a wide range or pre and post electrospinning modification approaches that broaden the range of functionalities that are possible hence making it possible to remove a broad range of contaminants, 4) there are several formats in which the nanofiber membranes can be arranged primary due to the flexibility of the supporting electrospinnable polymer backbone, 5) the electrospinning set up is relatively simple and it allows rapid prototyping as scaling up is simple (Chigome et al., 2011; Chigome and Torto 2011; Cheng et al., 2019; Shirazi et al., 2020). In our labs we have laboratory scale and pilot scale facilities of which our biggest pilot scale electrospinning unit allows the fabrication of a nanofiber membrane of 1 km length and 0.5 m width.

**FIGURE 1:**
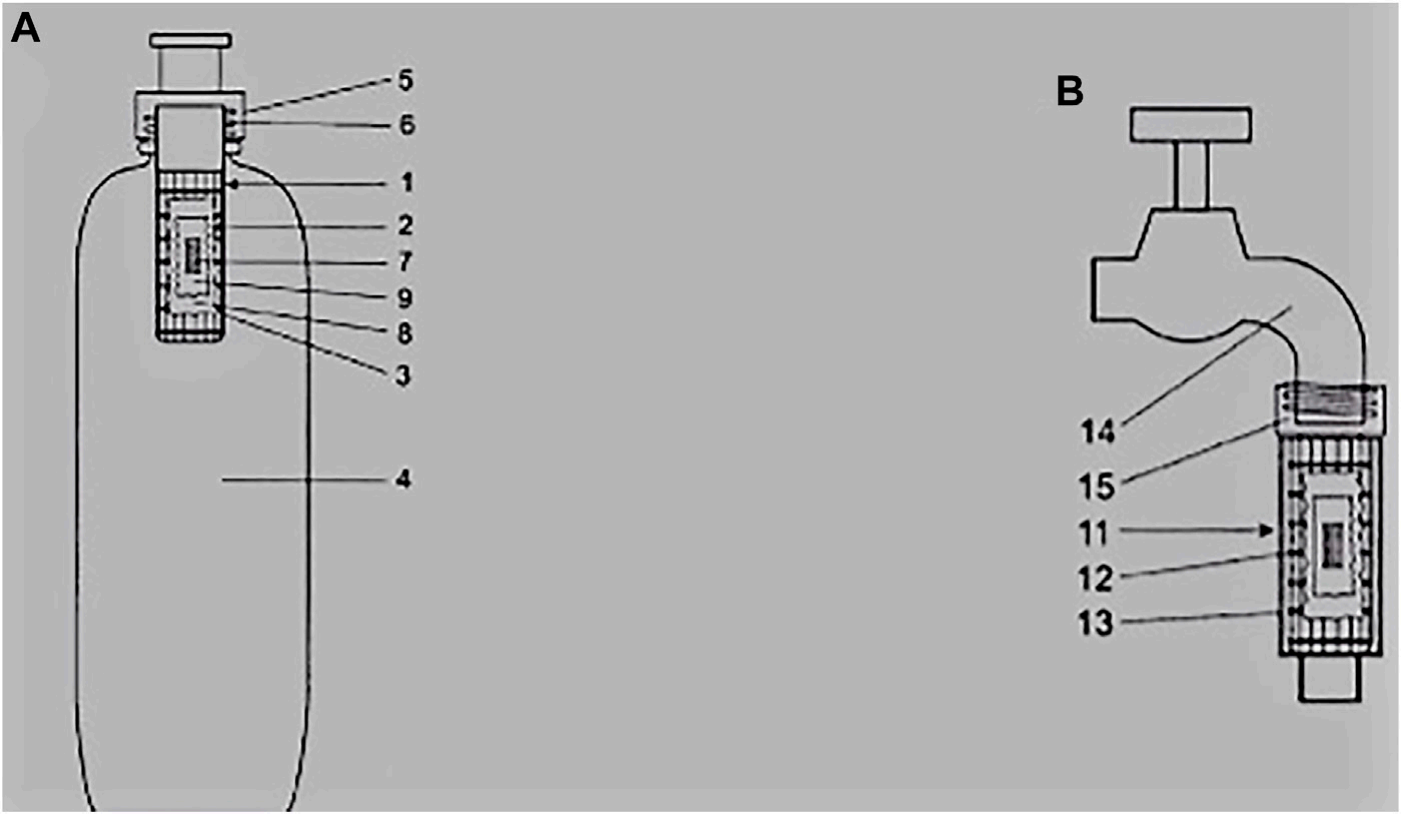
**(A)** water bottle and **(B)** tap outlet point of use water filters that rely on a teabag containing nanocomposite filter media 1 = cartridge housing, 2 = perforated housing, 3 = biocide/electrospun nanofiber layer, 4 = bottle, 5 = screw thread holder, 6 = threaded fitting, 7 = activated carbon, 8 = teabag, 9 = ion exchange resin,, 11 = rough surface housing, 12 and 13 = filter media housing, 14 = tap, 15 = threaded adaptor to fit to water supply (Dicks et al., 2012).

Figure 1 shows two types of electrospun fiber based point of use water filters, the first being a water bottle which brings an advantage of being portable while the second one is tap outlet water filter. The driving force for the water bottle is the pressure exerted by a hand during the process of squeezing while for the tap it is the typical municipal water pressure of about 1.5 bar. Both POU water filters in this example rely on the same filter media which is a teabag coated with a biocidal electrospun fiber membrane that contains granular activated carbon and an ion exchange resin (Dicks et al., 2012).

**FIGURE 2:**
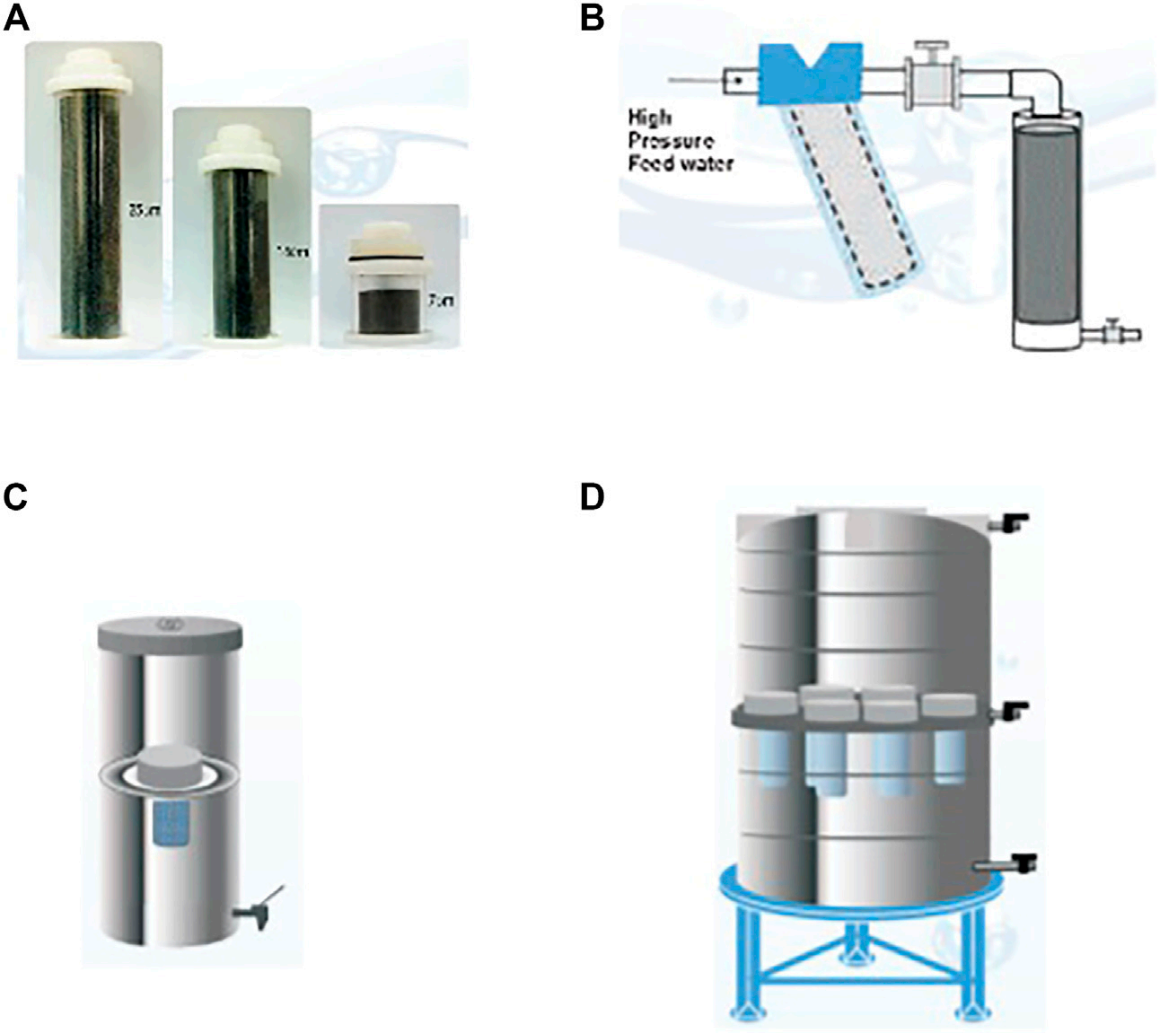
**(A)** granular filter media cartridges of different sizes for ease of integration into different filtration systems **(B)** cartridge connected to a high pressure online system like a borehole **(C)** granular cartridge incorporate into a kitchen top filter **(D)** multiple granular filter cartridges incorporated into a large tank for a community water filtration system (Pradeep 2012).

In order to vary the scale of POU water filters from household to community level with mainly an interest in gravity-based filtration systems, coarse granular filter media is packed into a cartridge format of varying sizes as shown in Figure 2. The shows the practicality of the concept of granular filter media of different compositions as scaling up and down is simplified.

**FIGURE 3:**
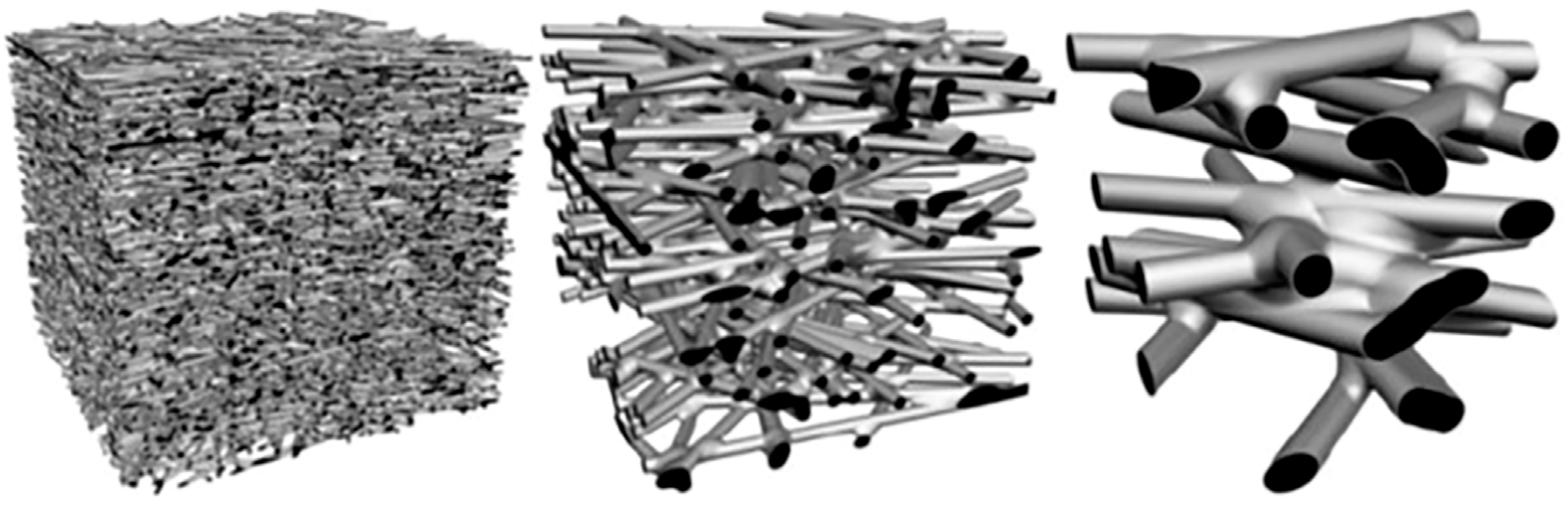
Illustration of the correlations between pore size and electrospun fiber diameter at a constant porosity of 80% in a fixed volume (Ma et al., 2011).

To help with the understanding of the electrospun nanofiber membrane properties, Figure 3 shows 3D simulated non-woven membrane models, having different fiber diameters, which were created to illustrate the relationship between the fiber diameter and the pore size. All the three membrane models are at a constant porosity in a fixed volume with a relative fiber diameter ratio of 1 :3:10.3D non-woven structure can be fully defined by only two parameters, the fiber diameter and the bulk porosity, and has no other adjustable parameters. Thus, the pore sizes can be adjusted by controlling the fiber diameter of the electrospun membrane, allowing them to satisfy the requirements for various microfiltration and ultrafiltration applications. This makes it possible to have high flux gravity driven filtration with the removal of bacteria of average size 200 nm by two mechanisms, size exclusion or bacterial conformational change (Abrigo et al., 2015). The multiple pathways that exist as a result of fiber diameter reduction illustrate the increased filtration efficiency due to increased available fiber surface and tortuous flow. The model therefore illustrates the benefits of having filter media in the electrospun nanofiber format where continuous intertwining fibers provide a effect membrane network for water filtration.

**FIGURE 4:**
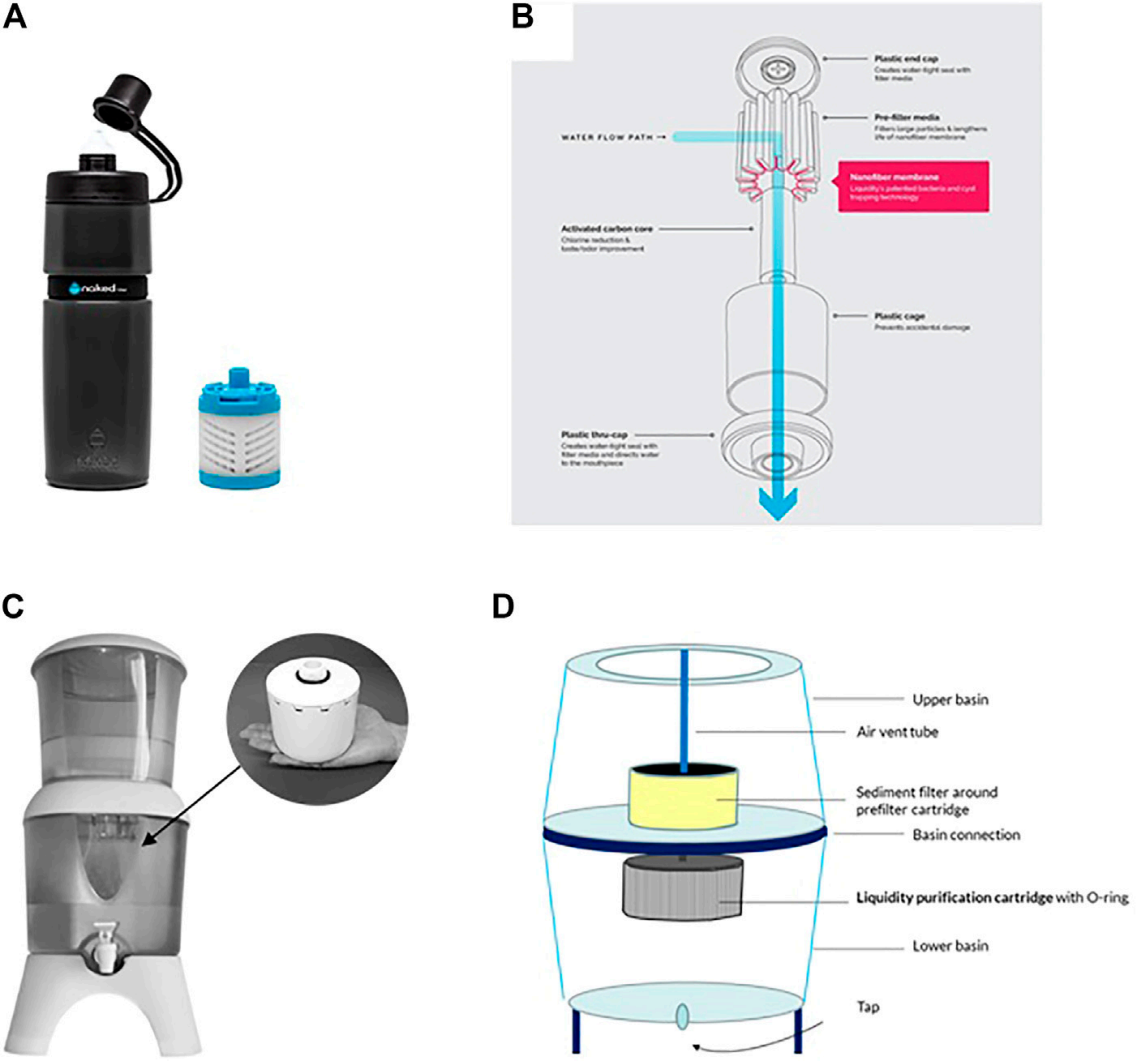
**(A)** bottle type nanofiber water filter (insert replacement cartridge) that has a filtration capacity of 75 L **(B)** components of the filter **(C)** Counter top nanofiber water filter (insert replacement cartridge) that has a filtration capacity of 1500 L **(D)** components of the filter (Liquidity Corpopration 2014).

Figure 4 shows two types of POU water filtration formats derived from electrospun nanofibers demonstrating the practical usefulness of the filter media as a small gravity driven cartridge can filter 1,500 L of water.

## 2 Materials for Point of Use Water Filtration Media

Chitosan has received greater attention in water purification due to presence of large number of^–^NH_2_ and OH^−^groups. The free amine function of chitosan gives it a better ability to chelate ions (Sudheesh et al., 2013). These chelating properties are of great interest for water treatment and particularly to recover metals present in contaminated water (Koshijima et al., 1973; Kyzas et al., 2009; Rajiv Gandhi et al., 2010).

The use of the metal oxides was to specifically harness their adsorption affinity towards arsenate and more importantly to achieve higher mechanical strength to produce granules of specific sizes instead of powder. Metal oxides that have been used for arsenic remediation include activated alumina, iron oxides, titanium oxides, zirconium oxides, cerium oxides and manganese oxides among other (Chen et al., 2014). The synergistic adsorption parameters of these oxides were harnessed in the form of a composite in order to increase the surface area of the metal oxides consequently increasing the number of adsorption sites and surface hydroxyl groups on the oxides.

Membrane technologies such as nanofiltration and reverse osmosis have found immense application in water purification. This is because membrane processes are capable of removing high concentrations of inorganic ions, dissolved solids and organic contaminants (Lakherwal 2014). However, membrane technologies are characterized by fouling and require high pressures for operations. This increases the cost of operation. The worst disadvantage of these technologies is that they can decrease mineral content in water, thus rendering the output water unsuitable for drinking (Pangarkar et al., 2011).

Nano-engineered materials can help in resolving water quality issues by using nanoparticles and nano-adsorbents. These materials have been proven to be efficient, strong and more affordable than other water decontamination technologies (Qu et al., 2013). Some of these products include nonporous filters such as ceramics, clays adsorbents, zeolites, dendrimers, metal-containing nanoparticles, carbonaceous and catalysts nanomaterials (Thakre et al., 2010; Ghauri et al., 2013).

Four major functional categories of nano-engineered materials are employed in water purification. To begin with, there are nanoparticles that have been tested and found to have antimicrobial properties such as silver nanoparticles. Next, there are nano-catalysts whose primary application is to reduce pesticides and other organic toxins. Thirdly, there are nanoadsorbents which are commonly applied in removal of inorganic contaminants such as heavy metals. Lastly, there are classes that are used as filtering agents for instance in membranes (Guo and Tian 2013).

## 3 Nanocomposite Filter Media

Fabrication of water purification media has been carried out in our lab using metal oxide biopolymer nanocomposite. The material was synthesized using green approach. In this study we have focused on the removal of arsenate and fluoride at laboratory scale and fabrication of a point of use water filter. At the laboratory level we focused on batch and continuous mode. Factors that affect adsorbent performance (pH, adsorbent dose, kinetics and co-existing ions) were investigated.

### 3.1 Nanocomposite Synthesis

The granular composite was synthesized using a combination of aluminium sulphate decahydrate, titanium (IV) oxysulfate, Zirconium oxychloride octahydrate and iron oxyhydroxide and chitosan. About 1.3 g of chitosan was dissolved in 100 ml of 1% HCl and mixed using a magnetic stirrer at 400 revolutions per minute (RPM). The mixture was left to incubate for 12 h. Exactly, 25 ml of chitosan solution was measured and incubated for 5 min and to it 10 ml of 0.5 M Al_2_(SO_4_)_3_.18H_2_O was added and stirring continued for 30 min. Next, 4 ml of 0.25 M titanium (IV) oxysulfate was added. Thereafter, 4 ml of 0.25 M ZrOCl_2_·8H_2_O was added. After 1 h of continuous stirring, 7 ml of 1 M FeCl_3_.6H_2_O was added and mixing continued for 30 min. About 3.6 g Na_2_SO_4_ was then added in one step to the incubating mixture. The mixture was precipitated and the pH brought to 6.5 by slow addition of 2 M NaOH. The final mixture was further left to incubate for 12 h at ambient temperature. The next step was to carry out vacuum filtration to obtain the gel. The subsequent gel was washed with copious amount of water in order to remove excess soluble salts. The gel was dried at room temperature for 12 h. Finally, the material was crushed into specific sizes (52 × 72 μm) and was ready for various adsorption studies.

### 3.2 Adsorption Experiments

As (V) stock solution (1,000 ppm) was prepared by dissolving sodium arsenate in 1 L distilled water and was preserved with 0.5% trace metal grade HNO_3_. All other arsenic solutions were prepared by dilution from the stock solution. The incubation time was chosen to be 2 h for all the experiments. Amount of the adsorbent required was added into the water samples and agitated at 210 RPM. Afterwards, water samples were decanted and centrifuged at 4000 RPM for 5 min and then filtered using 0.22 μm 6,6 Nylon membranes. The sample were then prepared by taking 950 µL of the water sample and digested by adding 50 µL HNO_3_. The effect of adsorbent dose on the sorption of As (V) on the adsorbent was carried out using 1,000 ppb as the initial arsenic ion concentration. The dosage was varied from 5 to 200 mg. To examine the effect of contact time, the adsorbent was exposed to As (V) in the range of 1–180 min. The effect of pH, adsorption experiments were conducted in the pH range of 4, 5, 6, 7, 8, 9 and 10. The initial arsenic concentration was taken to be 1,000 ppb. The pH adjustments were achieved using 0.1 M HCl and 0.1 M NaOH. The interference of competing ions such as Cl^−^, NO_3_^−^, SO_4_^2-^, Na^+^ SiO_3_^2-^, K^+^, Ca^2+^, Mg^2+,^ HCO_3_^−^, CO_3_^2-^ and HCO_3_^−^ and CO_3_^2-^ were investigated to evaluate their effect on adsorption efficiency. The effect of the initial As (V) concentration on the adsorption capacity of the nanocomposite material was tested in the range of 1–200 ppm with an adsorbent dosage of 50.0 mg. Maximum adsorption capacity of the nanocomposite was evaluated at concentrations ranging from 1 to 200 ppm were used.

### 3.3 Prototype Filter Fabrication

A prototype filter was designed and fabricated after the performance of the batch adsorption experiments was established. Approximately 60.0 g of the nanocomposite material of particle size 72 um was packed in water purification cartridge measuring 35 mm in diameter and 10 mm in height. The filter was assembled in antigravity manner.

### 3.4 Material Characterization

**FIGURE 5:**
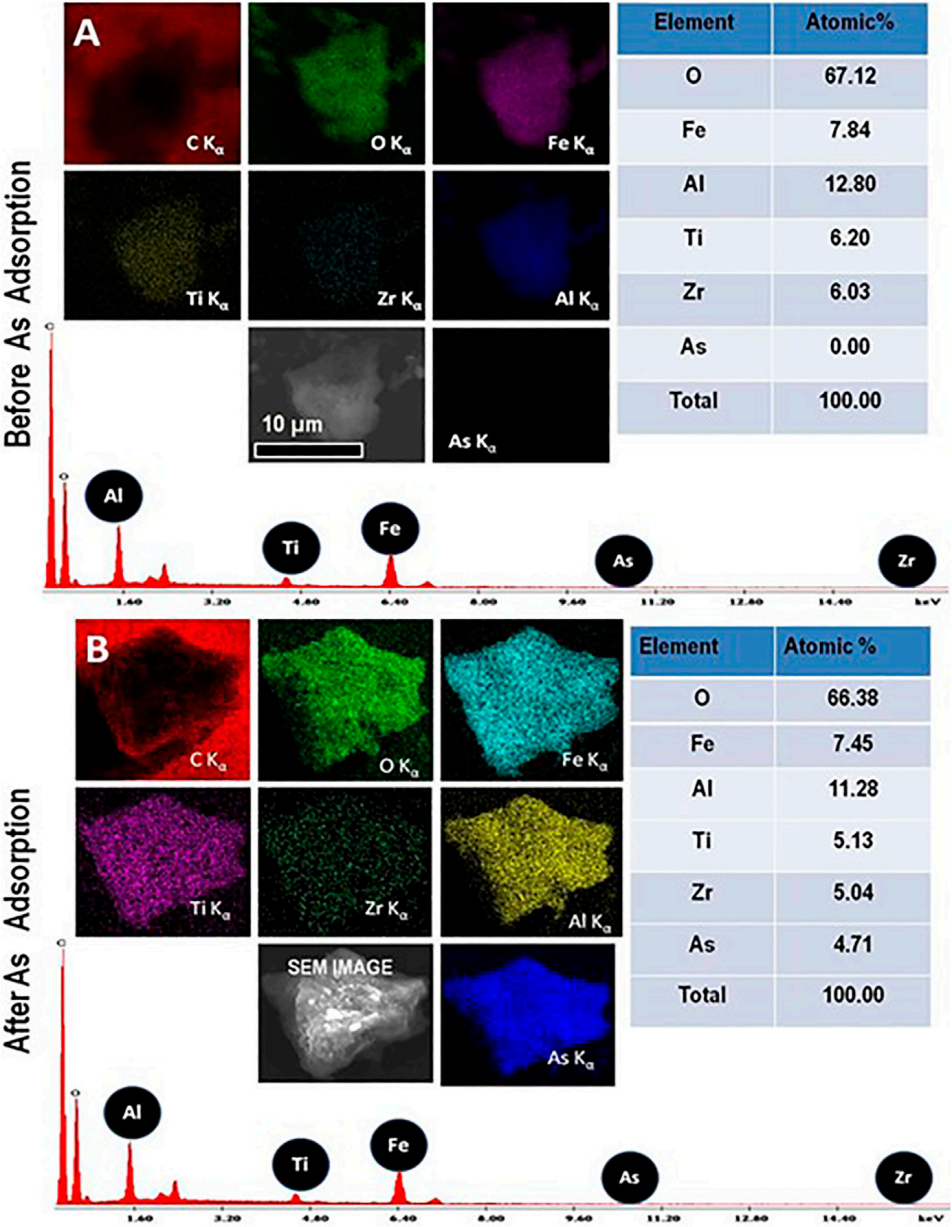
Elemental X-ray images (SEM-EDAX) of **(A)** the nanocomposite before exposing to As solution. **(B)** nanocomposite after exposing to As solution.

SEM-EDAX analysis was done on the nanocomposite material’s interaction with arsenic in form of As (V). The EDAX spectrum indicates that all the principal elements are present. Figure 5 presents an EDAX spectrum before (a) and after (b) adsorption.

The elemental composition maps confirm that the distribution of arsenic is homogeneous. It is clear that the level of arsenic before adsorption was 0.00% by percent atomic. However, after adsorption, the adsorbed content of Arsenic is recorded to be 4.71 atomic percent. This confirms the interaction of the adsorbent and arsenic.

SEM-EDAX analysis was done on the nanocomposite material’s interaction with arsenic in form of As (V). The EDAX spectrum indicates that all the principal elements are present. Figure 5 presents an EDAX spectrum before (a) and after (b) adsorption.

**FIGURE 6:**
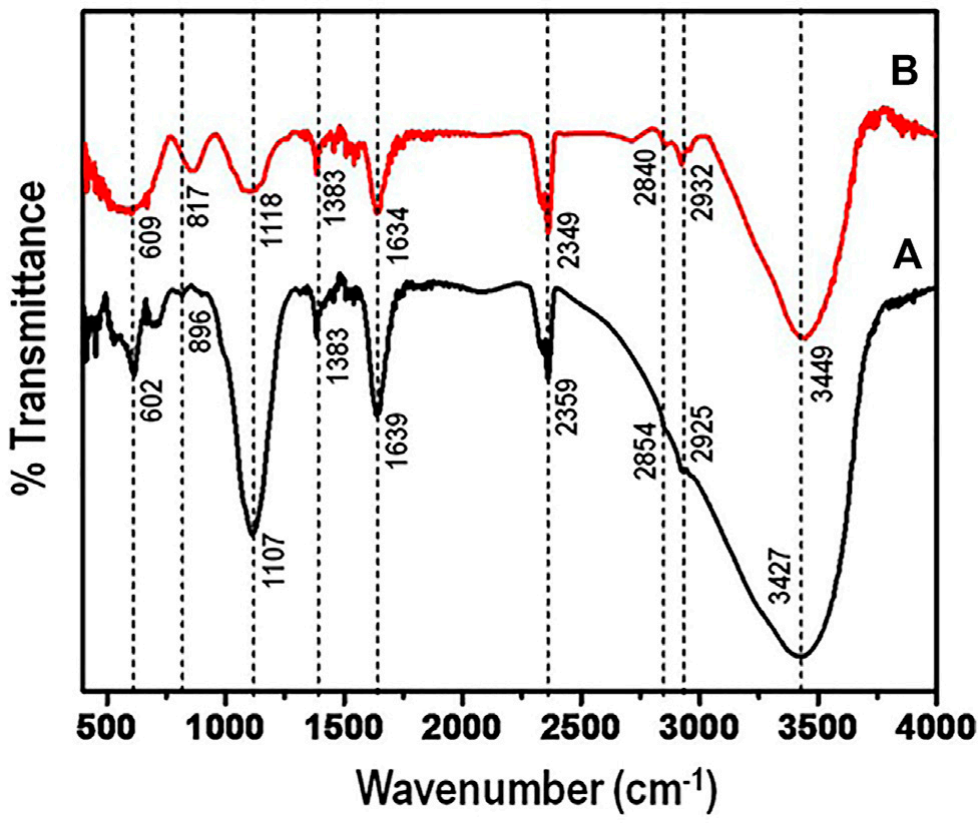
(FTIR) spectrum of aluminium-titanium-zirconium-iron oxyhydroxide-chitosan composite **(A)** along with the composite after adsorbing arsenic **(B)**.

To establish the functional groups that account for the adsorption of arsenic, FTIR spectra of the nanocomposite before and after adsorption was measured. Figure 6 shows the FTIR spectrum of aluminium-titanium-zirconium-iron oxyhydroxide-chitosan composite before exposure to arsenic solution (a) and after adsorbing arsenic (b).

The frequency band at wavenumber 1107 cm^−1^ is indicative of bending vibration of hydroxyl groups on the metal oxides. The peak intensity at 1,107 cm^−1^ after adsorption, decreased indicating arsenic adsorption. Generally, after exposing the nanocomposite to arsenic ion solution, it is notable that the intensity of the peaks reduced significantly. This phenomenon has been observed in other various studies, where the peak intensity reduces after adsorption (Qiusheng et al., 2014). However, two new broad peaks appear in the region of 600–1,000 cm^−1^ when compared to the spectra before arsenic adsorption. This might correspond to the adsorbent - arsenic interactions.

In addition, the following peaks showed shifts: 3,742 cm^−1^ to 3,749 cm^−1^, 2,935 cm^−1^ to 2,932 cm^−1^ and 2,854 cm^−1^ to 2,840 cm^−1^. However, there is a significant shift of peak 896 cm^−1^to 817 cm^−1^, with the intensity of the peaks increasing. From the FTIR studies, the results suggest that the replacement of the metal-hydroxyl groups by arsenic plays a significant role in the adsorption of arsenic contaminant.

The surface area of the aluminium-titanium-zirconium-iron oxyhydroxide-chitosan composite was investigated using BET. The specific surface area of the nanomaterial was recorded using Micromeritics ASAP 2020. Samples were degassed at 200°C for 4 h under vacuum and analyzed at 77 K with ultra-high pure nitrogen gas. It was found that the nanocomposite recorded a specific surface area of 153 m^2^g^−1^. The increased surface is most likely, among other factors, responsible for the enhanced adsorption capacity for both fluoride and Arsenic contaminants compared to other adsorbents but slightly lower than the one reported by Thakre and co-investigators who did similar study on chitosan based mesoporous Ti-Al binary metal oxide and posted a surface area of 323.8 m^2^g^−1^ (Thakre et al., 2010).

### 3.5 Batch Adsorption Studies

#### 3.5.1 Effect of Adsorbent Dosage

**FIGURE 7:**
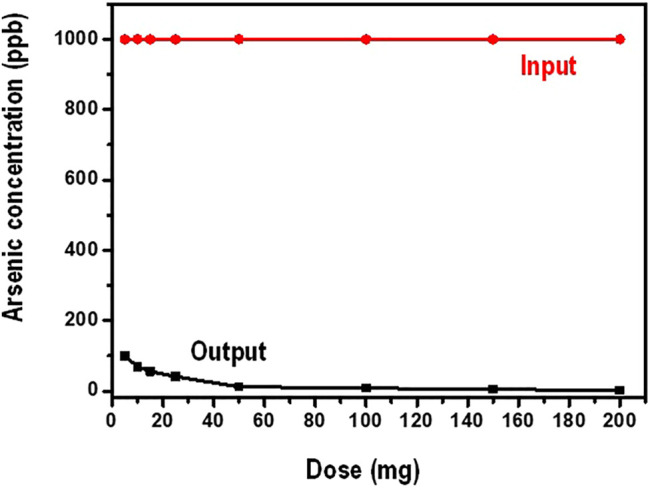
Residual arsenate concentration as a function of adsorbent dose.

In this investigation, the working volume of water was kept at 100 ml while the amount of the nanocomposite (dosage) was varied between 5 and 200 mg. The initial concentration arsenate was maintained at 1,000 ppb. Figure 7 shows data on the influence of the amount of the adsorbent dosage on the sorption of arsenate.

It is observed from Figure 7 that, increasing the amount of the adsorbent, the equilibrium concentration of arsenate decreases gradually. Generally, adsorption is greatly influenced by the number of the active binding sites present for the contaminant (adsorbate) to occupy. In lower adsorbent doses the available binding sites are minimal hence lower adsorption capacity. In higher adsorbent amounts, the presence of binding sites is high for the adsorbate to bind thus high adsorption capacity. This kind of observation is responsible for increased arsenate adsorption at higher a mounts of the adsorbent. In addition, increase in the amount of the adsorbent leads to increase in the number of active sites of adsorption. Moreover, increasing the dosage translates to increasing the surface area of the adsorbent (Sokker et al., 2011).

#### 3.5.2 Effect of Contact Time

**FIGURE 8:**
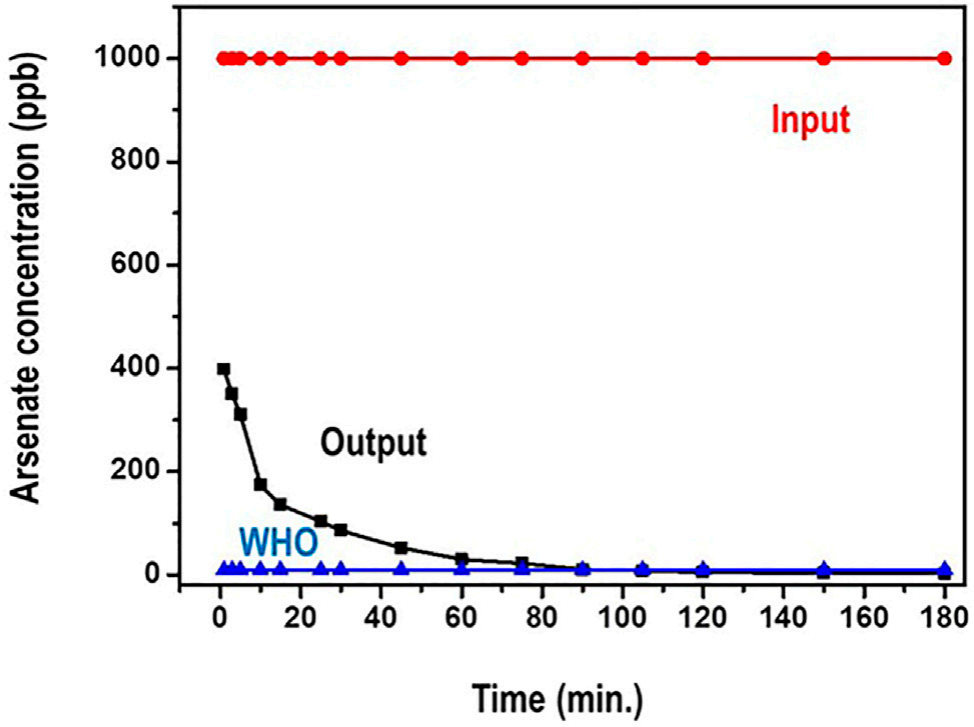
Residual arsenate concentration as a function of time.

The impact of contact time on the adsorption of arsenic species on the adsorbent was studied in the interval of 1–180 min. This was particularly to evaluate the equilibrium time for maximum adsorption. 1,000 ppb concentration of arsenate solutions was used. The amount of the adsorbent was maintained at 100 mg. Figure 8 presents results on the time dependence of arsenate adsorption on the nanocomposite.

From Figure 8, the arsenate uptake on the adsorbent was very rapid and over 95% adsorption was achieved within the first 20 min. Further, the sorption reached equilibrium at about 90 min. During the period after the 90 min, there was no significant increase in arsenate adsorption that was registered as the amount of time increased. Actually, it is noted that the concentration of arsenate reduces from 1,000 ppb to less than 10 ppb (WHO recommended limit) in about 75 min.

Initially, the fast adsorption could be ascribed to the higher concentration gradient and the availability of more adsorption sites (Qiusheng et al., 2015). After equilibrium was attained, it is possible that the available binding sites were strenuous to binding due to the repulsive forces between the solute molecules and the bulk phase of the adsorbent. Such similar phenomenon has been observed by Sokker and co researcher (Sokker et al., 2011).

#### 3.5.3 Effect of pH

**FIGURE 9:**
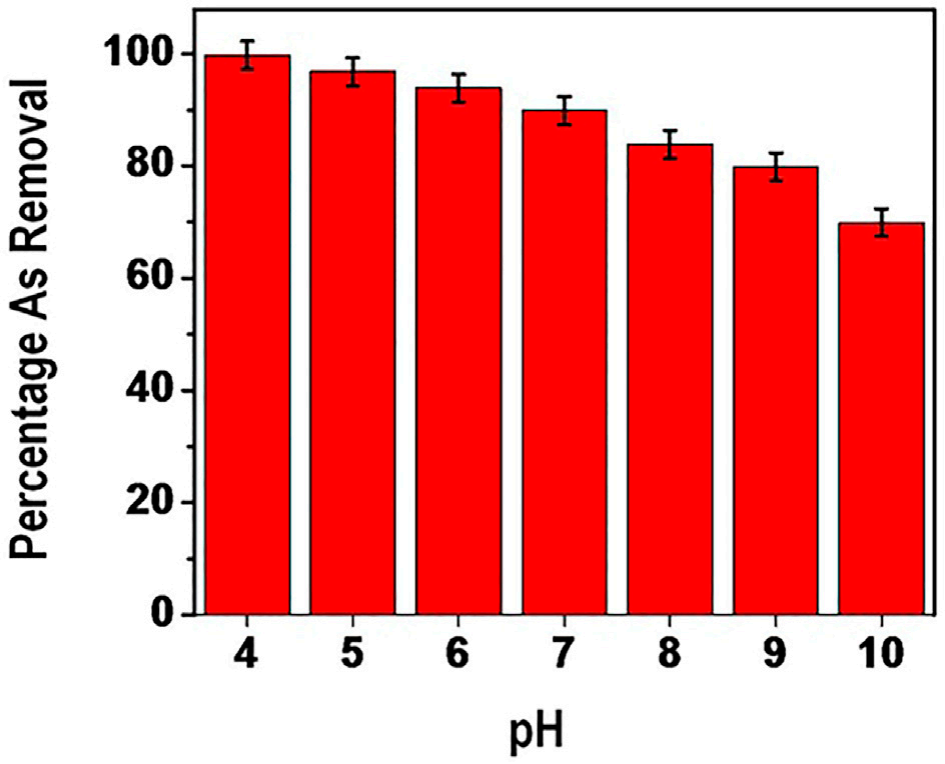
percentage removal of Arsenate as a function of pH.

The influence of pH was investigated in the range of 4–10 visa Vis the performance of the adsorbent. Figure 9 shows results the influence of initial solution pH on arsenate adsorption.

Percentage removal of arsenate was reduced from about 99% to about 69% when the pH was increased from 4 to 10. This is explained by the fact that there is minimal repulsion in the wide range pH. Specifically, percentage removal was above 90% in the pH range of between 4 and 7. This can be attributed to the stability of H_2_AsO^−^_4_ at pH between 2 and 7 (Table 1) hence existence of high amounts ions. However, a gradual decline trend on arsenate adsorption is observed after pH 7.

**TABLE 1:** pH stability for different species of Arsenate.

Arsenate Species	pH Stability
H_3_AsO_4_	<2
H_2_AsO^−^_4_	2–7
HAsO_4_^2-^	7–11
AsO_4_^3-^	>12

The above observation can be explained by the influence of pH on the speciation of arsenic in solution whereby beyond neutral pH arsenate particularly HAsO_4_^2-^ ions are prevalent compared to H_2_AsO^−^_4_. It should be noted that HAsO_4_^2-^ ions have got less affinity for the adsorbent compared to H_2_AsO^−^_4_ thus the decline adsorption capacity. The table below presents stability of various species of arsenate at different pH levels.

It is evident from Table 1 above that when equilibrium pH increases the concentration of multivalent species increases in the solution and consequently is not easily adsorbed onto the nanocomposite. Usually As (V) mainly exists as H_2_AsO^−^_4_ in the pH range of 3 and 6. In the pH range of 8 and 11 As (V) occurs as HAsO_4_^2-^ (Rajiv Gandhi et al., 2010). In the pH range of 4–8 it could be possible that the nanomaterial surface gains negative charge hence high specific adsorption of As (V) onto the nanomaterial. The relatively high percentage (above 90%) of arsenic removal up to about pH 7 can be linked to the high degree of electrostatic attraction between the As (V) and the positively charged surface of the nanocomposite.

From the amphoteric theory on dissociation it is opined that as pH increases there is a decrease in adsorption of As (V) due to the decline in electrostatic interaction between the nanocomposite and As (V). Similar observation has been reported by (Nabi et al., 2009). In their findings they found out that the adsorption efficiency of As (V) using Titanium nanoparticles decreased above pH of 7. Further, there is increased competition for the active site from OH–ions hence decrease in arsenic adsorption. This is in agreement with what has been documented by Yu et al. (2015).

#### 3.5.4 Effect of Competing Ions

**FIGURE 10:**
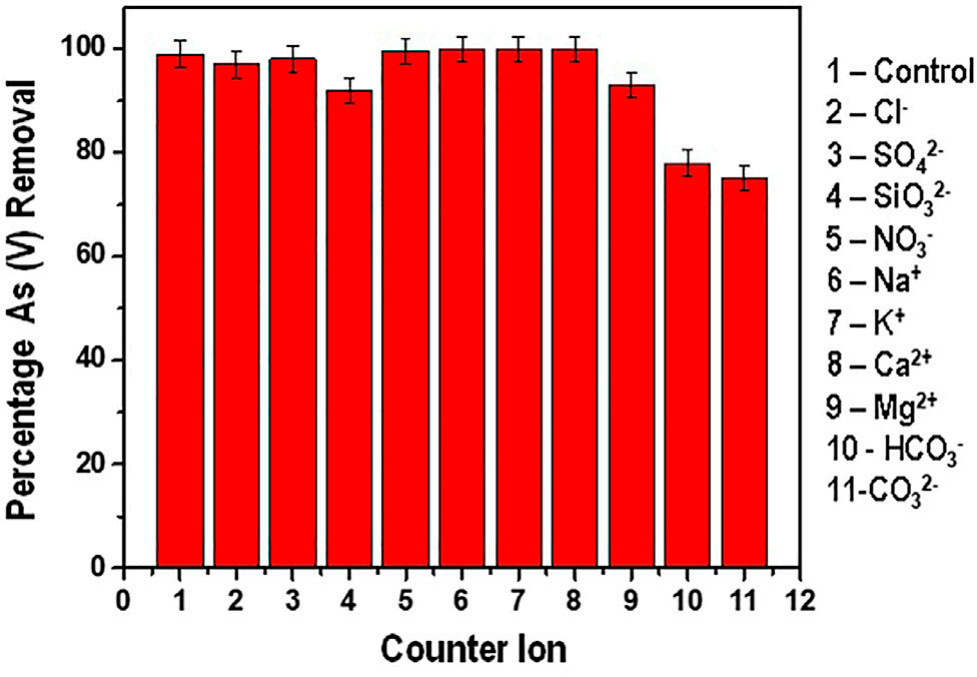
Percentage Arsenic (V) removal in presence of various counter ions.

The presence of various dissolved ions in water presents a challenge of these ions competing for the active sites with the targeted adsorbate. Their presence in water affects the overall performance of the adsorbent. The effect of the various ions present in water and their effect on As(V) adsorption was studied and the results are presented in Figure 10.

From Figure 10 it is depicted that the presence of Cl^−^, SO_4_^2-^, NO_3_^−^, Na^+^, K^+^, Ca^2+^ and Mg^2+^ did not present significant interference with the adsorption of As (V). In the presence of these competing ions, the reduction efficiency recorded was above 95%. Similar results have been reported by Ghosh and co-investigators in their work on Arsenate removal using iron and magnesium salts. In their findings, they observed that Cl^−^ and NO_3_^−^ ions had inconsequential impact on the adsorption efficiency. Contrary to this study, the presence of SO_4_^2-^ ions had pronounced effect on the adsorption process (Ghosh et al., 2003).

The presence of SiO_3_^2-^, CO_3_^2-^ and HCO_3_^−^ had a significant negative effect in the adsorption efficiency. The effect of SiO_3_^2-^ on the adsorption efficiency can be attributed to the competition for the binding site with the arsenate species as described by Tresintsi and co-researchers (Tresintsi et al., 2012). Genc-Fuhrman and his co-author have equally documented that silicates can reduce the adsorption efficiency of arsenic from 100% to about 60% in the pH ranges of drinking water (6.5–8.5) (Genç and Tjell 2003).

The CO_3_^2−^and HCO_3_^−^ ions had the most pronounced negative effect with the adsorption efficiency reducing to less than 80% (reduction from an initial concentration of 300 ppb to an average of about 60 ppb). This observation can be well explained by the fact that when carbonate and hydrogen carbonate ions are present in water, the pH increases significantly. As earlier discussed on the effect of pH on As (V) adsorption, it therefore means that the drop in adsorption efficiency is due to the increase in pH which further leads to the increase in the electrostatic repulsion between the predominant HAsO_4_^2-^ species and the negatively charged surface of the adsorbent at pH between 7 and (Saha and Sarkar 2016).

#### 3.5.5 Effect of Arsenic Concentration

**FIGURE 11:**
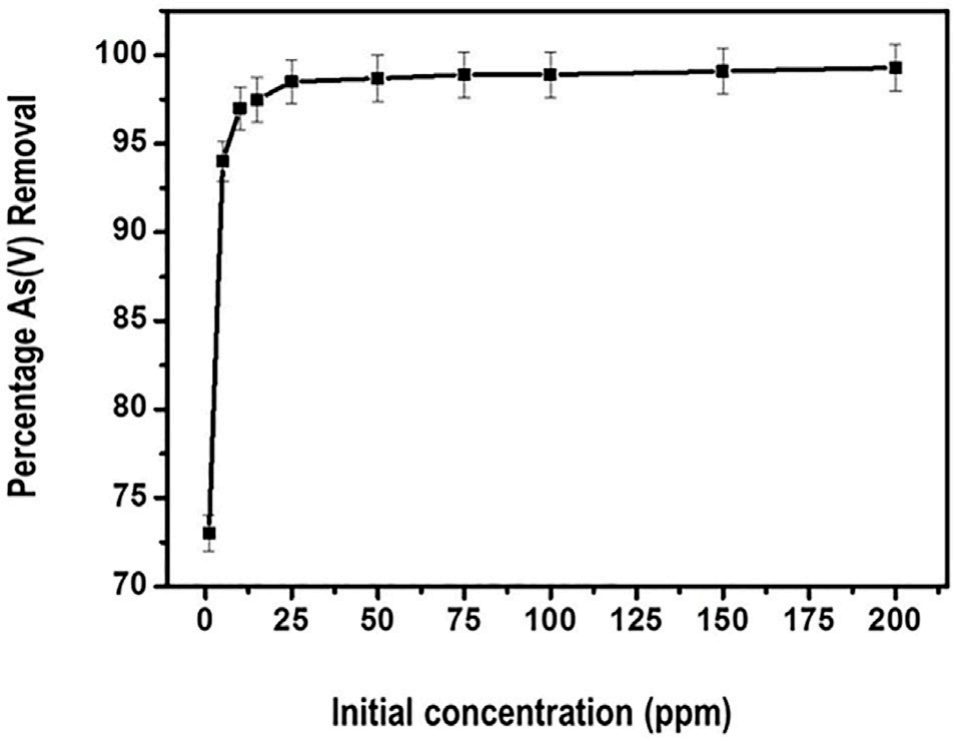
Adsorption as a function of initial arsenate concentration.

The influence of the initial As (V) concentration on the reduction efficiency was tested in the range of 1–200 ppm. The results are presented in Figure 11.

From Figure 11, generally the removal percentage of arsenate by the adsorbent initially increases with the increasing initial concentration of arsenate. Further, the optimum percentage removal level of about 98% is attained at 50 ppm arsenate concentration. Thereafter a plateau curve is noticed indicating attainment of equilibrium. This kind of observation could be explained by the fact that the availability of binding sites for adsorption remained fairly constant for the fixed amount of the nanocomposite.

The increase in adsorption efficiency as concentration of the adsorbate increased can be attributed to the decline in resistance of arsenate from the solution. This observation is in consistent with a similar study carried out by Roy and co-researchers. In their study, they posted an increase in arsenate adsorption when the concentration was increased to about 85.6%. Thereafter the percentage of arsenate reduction showed little increase (Roy et al., 2013).

#### 3.5.6 Adsorption Isotherm Studies

**FIGURE 12:**
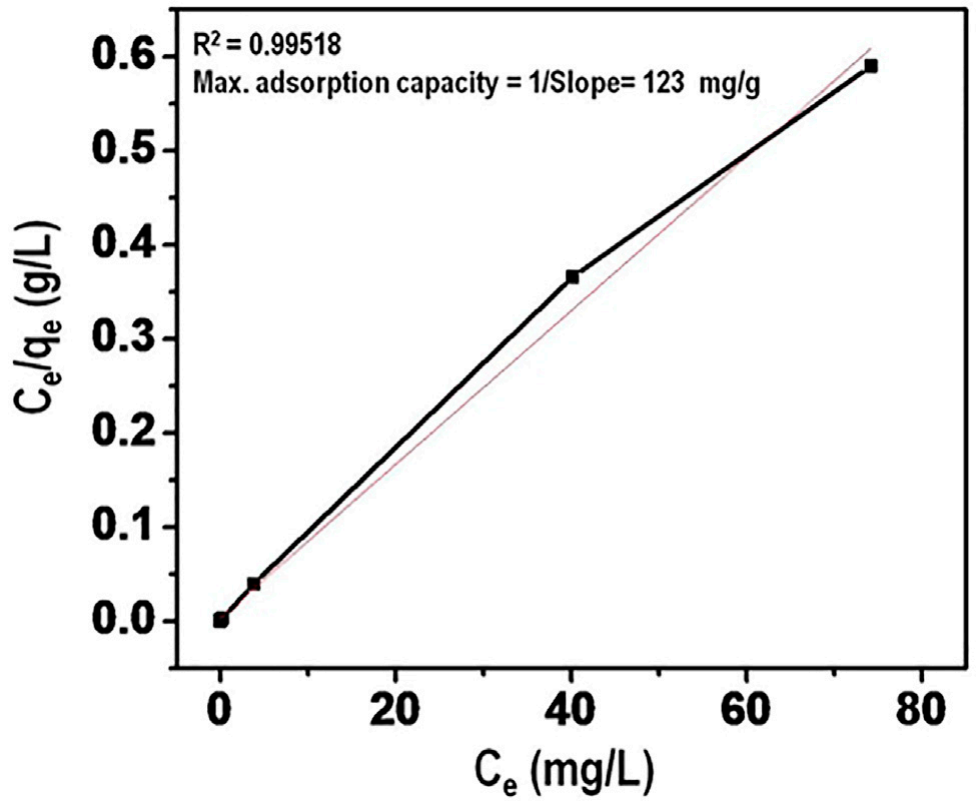
Langmuir adsorption isotherm for Arsenate ion adsorbed (mg/g).

Maximum arsenate adsorption capacity was evaluated using Langmuir adsorption isotherm. The data is presented in Figure 12. The linear plot of Ce/q_e_ versus C_e_ in addition to high value of correlation coefficient provides that Langmuir isotherm provides a better fit for the equilibrium data. The Langmuir capacity (mg/g) of the nanomaterial for arsenate adsorption was calculated to be 123 mg/g. This can be linked to the inherent structure of the nanomaterial which provides for generation of effective sites of adsorption. This value is higher than those reported in previous literature.

#### 3.5.7 Prototype Filter Performance Using Tap Water Laden With Arsenate

**FIGURE 13:**
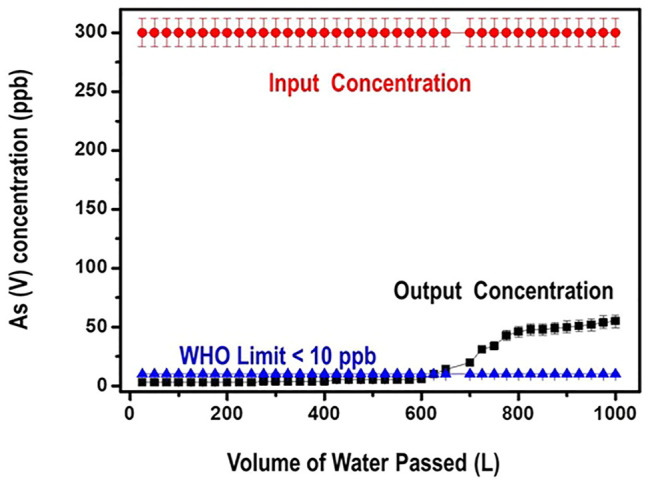
Performance data for Arsenate reduction in tap Water.

The filter’s performance against As (V) reduction efficiency was investigated with more than 1000 L of water at a flow rate of 10–15 ml/min. The input water had a concentration of 300 ppb of the As (V) contaminant. Output water samples were collected and analyzed for residual Arsenate after digestion with 5% HNO_3_. Data obtained from this study is presented in Figure 13.

Data from Figure 13 illustrates that arsenate contaminant was removed to below the WHO limit of 10 ppb for 650 L of tap water with only 60.0 g of the nanocomposite. It is equally noted from the graph that even after running 1,000 L of the arsenate contaminated tap water through the filter, still the performance of the nanocomposite was excellent such that the level of arsenate was reduced from 300 ppb to about 50 ppb. These results can translate to an adsorption capacity of close to 120 mg/g that is 3–8 times greater than the ones reported in literature.

### 3.6 Configurations for Powdered Filter Media

**FIGURE 14:**
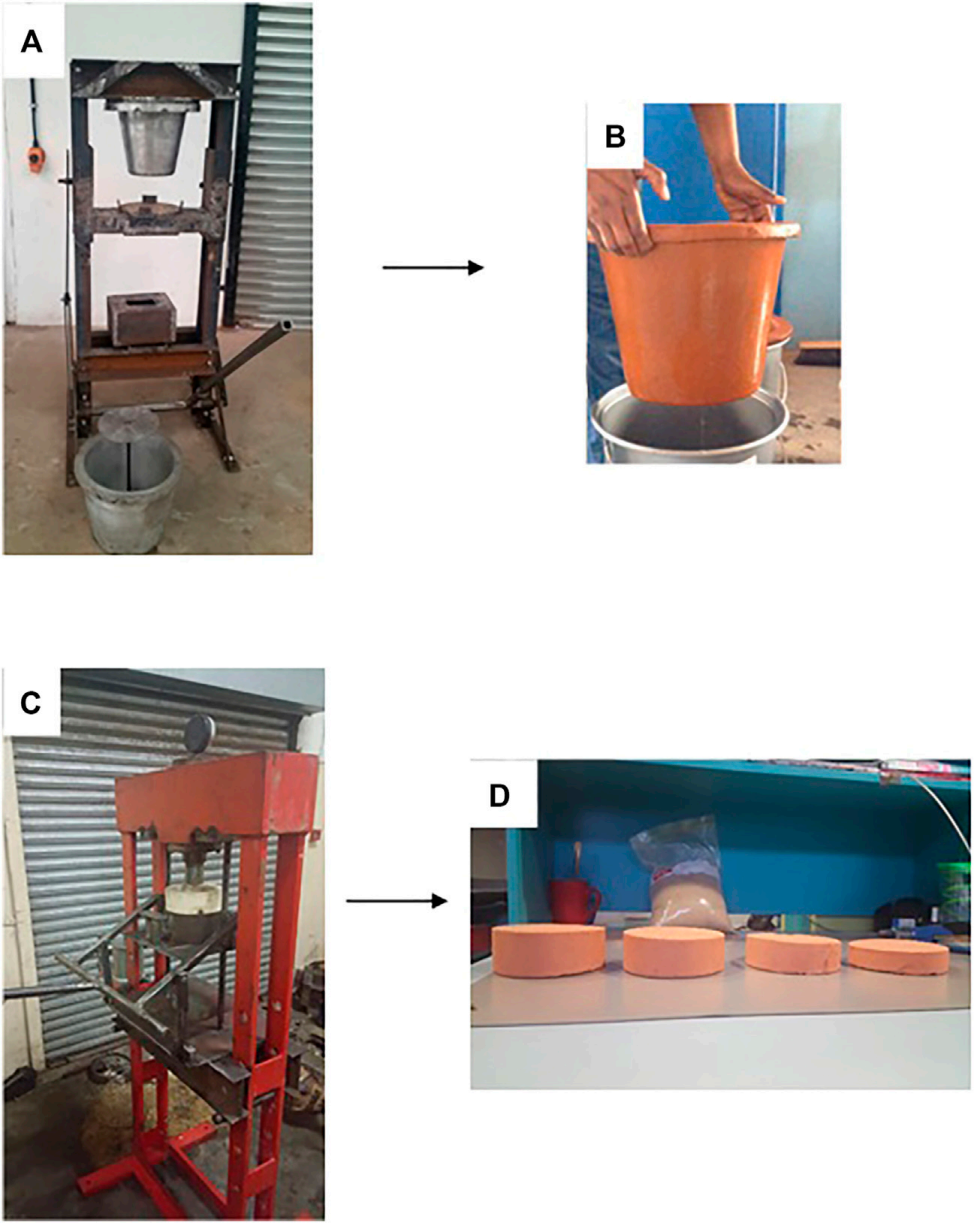
**(A)** Mechanical clay pot filter mould **(B)** Ceramic pot filter after heat treatment **(C)** Mechanical clay disk filter mould **(D)** Ceramic disk filters of different sizes after heat treatment.

In our labs, we explored the possibility of fabricating ceramic filter media derived from iron oxide rich clay, saw dust as a pore generator upon heat treatment and silver nanoparticles as the antimicrobial agent. We fabricated mechanical filter moulding devices for ceramic pot and ceramic disk filters (Figure 14). Such fabrication approaches could be taken advantage off for application to the fabrication of a variety of nanocomposite filter media particularly the disk configuration as it is less bulky as compared to the ceramic pot filter.

**FIGURE 15:**
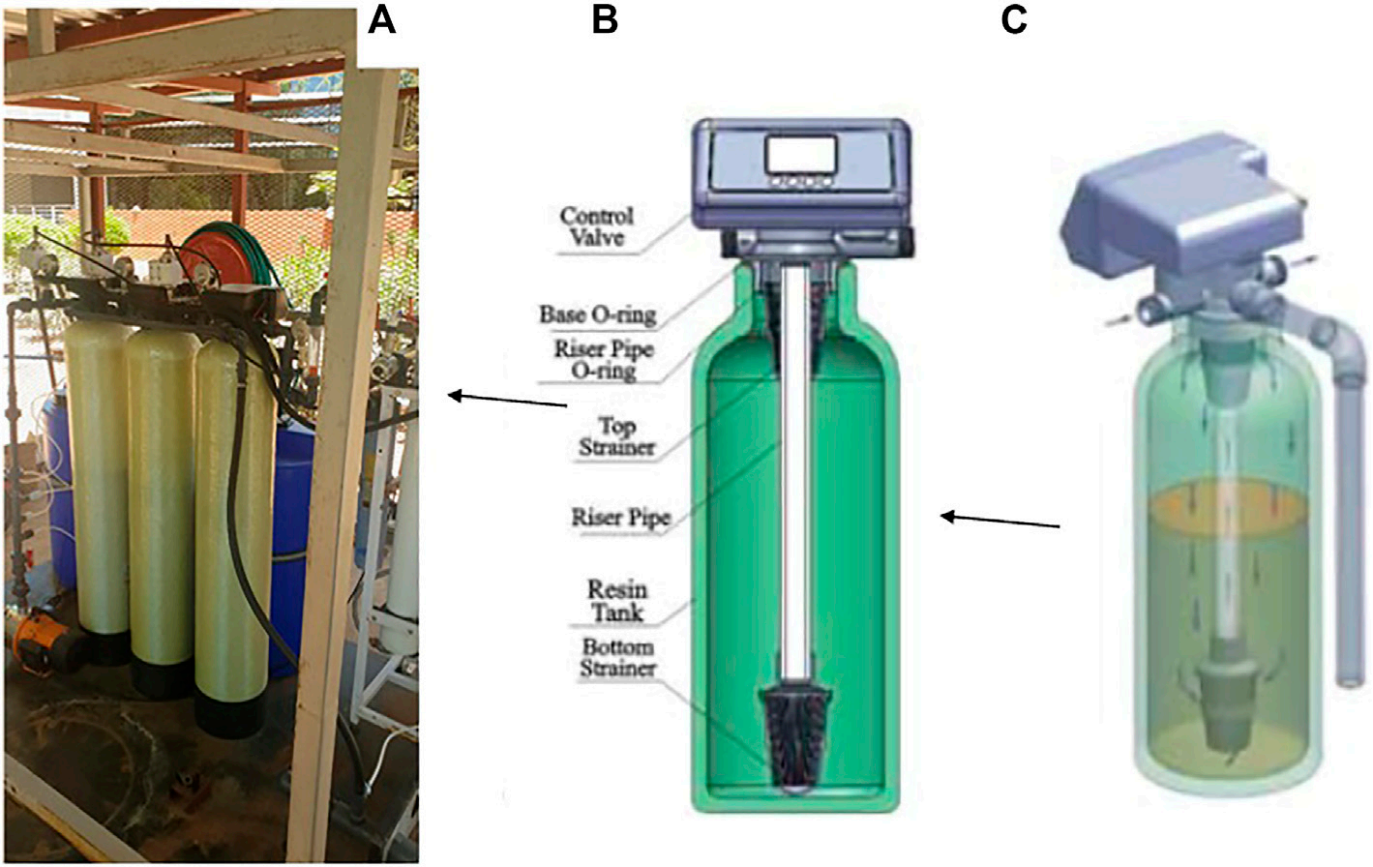
**(A)** A section of a 500 L/h pilot plant filtration system showing three stages of granular based filtration media **(B)** Components of each filter media vessel **(C)** Flow pathway of water during filtration.

Given the fact that in some instances there is a need for high flow rate multistage point of use water filtration systems to handle the complexities associated with different water sources, it is plausible to consider a configuration for housing a range of nanocomposite filter media. Such systems would benefit a community, or a company set up with more than 300 employees. Figure 15 shows a pilot scale water filtration experiment station that we have at our labs. The system is currently configured to house three types of nanocomposite filter media within the holding vessels. On the basis of the power rating of the feed pump, it can produce more than 300 L/h of water. In order to allow the use of the pilot scale water purification system in areas that do not have access to electricity, it is also possible to configure it so that the pumps can be solar driven.

## 4 Conclusion and Future Direction

The development of filter media for POU water filters is an area of importance as it helps to solve the challenge of accessing cleaning drinking water in areas that are far away from municipal water purification systems. Affordability of the resultant water filtration systems is mainly determined by the cost of the filter media. The cost is viewed from the material fabrication which is lowered firstly by using low-cost raw materials of which exploring the use of locally abundant natural resources is plausible. Secondly, the cost of the filter media is lowered by fabricating large capacity filter media which prolongs the filtration lifespan before regeneration of filter media replacement. Nanomaterials based filter media are seen as contributing to an increase filtration capacity mainly because of their large surface area. This implies that research efforts should be directed towards a better understanding of the application of nanotechnology-based fabrication techniques to produce efficient filter media derived from locally available raw materials.
